# Engineering dynamic cell cycle control with synthetic small molecule-responsive RNA devices

**DOI:** 10.1186/s13036-015-0019-7

**Published:** 2015-11-20

**Authors:** Kathy Y. Wei, Christina D. Smolke

**Affiliations:** Department of Bioengineering, Stanford University, 443 Via Ortega, MC 4245, Stanford, CA 94305 USA

**Keywords:** Synthetic biology, cellular devices, RNA engineering, cell cycle

## Abstract

**Background:**

The cell cycle plays a key role in human health and disease, including development and cancer. The ability to easily and reversibly control the mammalian cell cycle could mean improved cellular reprogramming, better tools for studying cancer, more efficient gene therapy, and improved heterologous protein production for medical or industrial applications.

**Results:**

We engineered RNA-based control devices to provide specific and modular control of gene expression in response to exogenous inputs in living cells. Specifically, we identified key regulatory nodes that arrest U2-OS cells in the G0/1 or G2/M phases of the cycle. We then optimized the most promising key regulators and showed that, when these optimized regulators are placed under the control of a ribozyme switch, we can inducibly and reversibly arrest up to ~80 % of a cellular population in a chosen phase of the cell cycle. Characterization of the reliability of the final cell cycle controllers revealed that the G0/1 control device functions reproducibly over multiple experiments over several weeks.

**Conclusions:**

To our knowledge, this is the first time synthetic RNA devices have been used to control the mammalian cell cycle. This RNA platform represents a general class of synthetic biology tools for modular, dynamic, and multi-output control over mammalian cells.

**Electronic supplementary material:**

The online version of this article (doi:10.1186/s13036-015-0019-7) contains supplementary material, which is available to authorized users.

## Background

Synthetic biology aims to make cells reliably programmable by applying engineering principles to the design of genetic circuits to control and monitor cellular behavior [1–7]. One approach to engineering genetic circuits leverages existing cellular networks and interfaces synthetic control systems at key nodes in these networks to rewire signal processing and cellular decision making [8, 9]. In addition, recent advances in the field are leading to genetic circuits of higher-order complexity in mammalian cells [10–14], which may ultimately be used to engineer human cells for sensing conditions in the body, producing drugs or useful materials, or addressing disorders of sophisticated phenotypes, such as those linked to cell cycle and differentiation.

Cell cycle, in particular, plays a key role in human health and disease, including development and cancer [15]. Cell cycle processes produce dramatic changes in cell physiology, but are often ignored in studies. The creation of model human cell lines that can be easily and reversibly paused at specific cell cycle phases can be used to further cancer research, cell cycle research, and biotechnology in general. For example, heterologous protein production for industrial or medical applications is likely more efficient during G0/1 and G2, when fewer resources are dedicated to division [16]. In addition, signal processing in the self-renewal versus differentiation decision is potentially linked to the length of certain phases of the cell cycle [17]. Thus, better understanding and control over cell cycle progression may aid in more effective cell re-programming [17]. Further, tools for genetic manipulation in mammalian cells are typically too inefficient for viable gene therapy and limit current research. Studies have shown that homologous recombination is more active in S and G2 phases, both of which are relatively short in a normal cell cycle [18]. Thus, the ability to pause cells in S and G2 has the potential to increase the reliability of mammalian genetic integration techniques. In addition, cells temporarily paused in G1 have been shown to stably express transgenes from episomes, which may provide a strategy for gene therapy that does not rely on disrupting the genome [19].

Current methods for altering cell cycle progression rely on small molecule inhibitors or nutrient deprivation methods [20]. While effective, these approaches can present a number of drawbacks, including exhibiting specificity to a particular cell type, being broadly disruptive of cellular processes, and being limited in capacity to extend to different networks due to lack of genetic encoding. A genetically encoded system supporting control over cell cycle progression has the potential to address limitations with existing chemical approaches. Such a synthetic biology-based approach relies on (i) the identification of key regulatory nodes in native cell cycle networks and (ii) the integration of tailorable gene-control devices that can modulate the activities of these nodes in response to user-specified signals.

RNA-based gene-control devices are a class of gene-regulatory elements that have been used in microbes to humans cells for diverse cellular applications [6, 8, 12, 21–25]. Ribozyme-based gene-control devices are self-cleaving RNA regulatory elements [26], where cleavage can result in transcript degradation when placed in the 3′-untranslated region (3′ UTR) of the target gene of interest. Ribozyme devices generally couple an RNA aptamer [27, 28] to the ribozyme element such that ligand binding to the aptamer affects the cleavage activity of the ribozyme. Thus, ribozyme devices can function as ligand-responsive genetic switches or ribozyme switches, where ribozyme activity, and thus target RNA and protein levels, are modulated as a function of ligand concentration in the cell [29]. Ribozyme switches can be efficiently developed through computational or high-throughput screening methods [29–31], where switches prototyped in microbial systems can be readily transferred to mammalian systems with predictable results [32]. Small molecule-responsive ribozyme switches have been used to achieve drug-modulated control over complex phenotypes in mammalian cells, including viral replication [22, 33] and T-cell survival and proliferation [12].

Here, we demonstrate ribozyme switches that control the arrest of human cells in various phases of the cell cycle. Specifically, we identified key regulatory nodes that induce arrest of cells in G0/1 or G2/M phases of the cycle and tune the expression of the most promising key regulators to optimize the arrest phenotype. We coupled theophylline-responsive ribozyme switches to the identified key regulatory nodes and demonstrated drug-responsive control over cell cycle arrest in G0/1 or G2/M. These genetic cell cycle controllers are able to inducibly and reversibly arrest up to ~80 % of a population of cells in a chosen phase of the cell cycle and perform reliably over multiple experiments over a time frame of several weeks. More broadly, ligand-responsive RNAs represent a class of synthetic biology tools that are both genetically encoded and capable of regulating intracellular protein levels in response to user-specified molecular signals, and can be adapted for sophisticated control of complex processes in human cells.

## Results and discussion

### Identifying key regulatory nodes that arrest cells in G0/1

The human cell cycle is a complex sequence of carefully timed activities that ensures proper genome replication and cell division/segregation. The signaling networks controlling cell progression between the different phases are multi-component systems and interwoven with other processes in the cell to safeguard genomic integrity through divisions. Despite decades of research, our understanding of the underlying networks and processes in the cell cycle remains incomplete. One trend that emerges from the body of prior work in this field is that despite the large number of genes involved in each cell cycle transition, in practice, a very small set of key protein regulators function as nodes. The regulatory nodes act such that changes in the expression level of that node results in measurable population level changes in cell cycle progression.

Previous studies have demonstrated the existence of such regulatory nodes and have used these nodes to investigate the biochemical mechanism of the cell cycle [34, 35]. However, it is unclear to what degree a single key node is able to control cell cycle (in terms of percentage of cells in a population that respond). It is also unclear to what degree an overexpressed key node is able to overcome the large amount of transcriptional and posttranslational regulation that the protein is under through control systems endogenous to the cell. A truly effective node may need to eliminate some of the sites of regulation in the protein to better direct the control of cell cycle without being disabled by the endogenous programs.

We set out to identify key regulatory nodes for inhibiting progression of G0/1 to S in mammalian cells and to determine the degree of inhibition associated with this genetic regulatory strategy. The cDNAs for a panel of putative regulatory node proteins were overexpressed from a mammalian expression plasmid in U2-OS cells by transiently co-transfecting 3–3.75 μg of the individual plasmids encoding the expression of these candidate proteins with 1.13-1.5 μg of a plasmid encoding a GFP reporter for use as a transfection marker for a total of 4.5 μg transfected DNA per sample. The panel of proteins screened was comprised of candidates most likely to exhibit the desired activity based on a review of the literature and represented a sampling of non-modified proteins as well as modified proteins, designed to eliminate some aspect of endogenous regulation (Additional file 1: Table S1). Cells across the cell cycle phases from each sample were assayed three days after transfection via DNA staining with propidium iodide followed by quantification using flow cytometry (Additional file 1: Figure S1-S2). These assay conditions were selected to allow reliable quantification of a sufficient number of cells per sample and supported rapid screening for regulatory nodes that are promising for further investigation. A plasmid encoding the expression of a fluorescent protein (mCherry) served as a negative control that does not alter cell cycle progression.

**Fig. 1:**
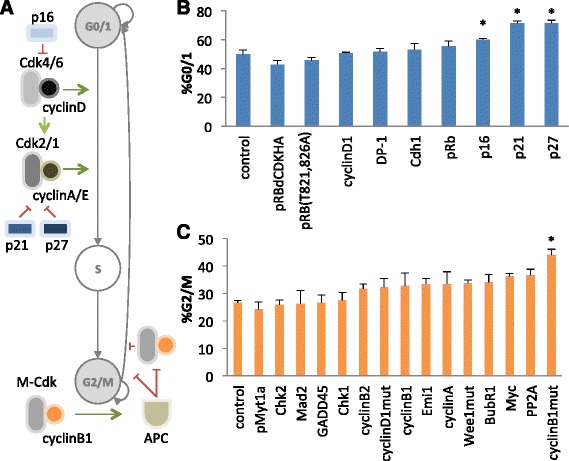
Screen to identify key regulatory nodes that produce cell cycle arrest in G0/1 and G2/M. **a** Schematic of the progression through the phases of cell cycle and a simplified representation of the identified key node function in cell cycle regulation. **b**, **c** Potential regulatory node proteins were overexpressed and the resulting cell populations were assayed for changes in the percentage of cells that were in G0/1 phase (**b**) or G2/M phase (**c**) relative to a negative control (i.e., control plasmid that does not alter cell cycle progression). *, *p* < 0.05. Cells were transiently co-transfected with 3–3.75 μg of the plasmids encoding the expression of these candidate proteins and 1.13–1.5 μg of a plasmid encoding a GFP reporter. Error bars represent standard deviation across biological triplicates

The data from the screening assay indicate three regulatory nodes for inducing a G0/1 arrested state in U2-OS cells - p16 (~60 % in G0/1), p21 (~71 % in G0/1), and p27 (~71 % in G0/1) (Fig. 1a, b). The unmodified cell cycle program (i.e., mCherry expression) results in ~50 % of cells in G0/1 under the same conditions. Compared to using double thymidine block, a commonly used small molecule based method for arresting cells in G0/1 and early S phase, which arrests approximately 86-95 % of cells (Additional file 1: Figure S3) [36], the identified regulatory nodes are less effective, but still represent an appreciable amount of modification of the cell cycle (Additional file 1: Figure S3). Thus, a single protein in a complex network, which contains at least 142 proteins directly annotated by the Gene Ontology project as being involved in the “G1/S transition of mitotic cell cycle” in *Homo sapiens* (or ~0.7 % of the network), controlled up to 42 % of the cells that were previously escaping G0/1 arrest (AmiGO 2.2.0) [37]. No modifications to the identified regulatory node proteins were necessary for their activity in inhibiting cell cycle progression. It is of note that these conclusions are specific to U2-OS cells at three days after transfection and not necessarily generalizable to other cell lines or other time points in a transient transfection assay.

The identification of p16, p21, and p27 as key regulators of the G0/1 to S transition is supported by their roles in the cell cycle network (Fig. 1a). Specifically, p16 (INK4a) is a well-studied inhibitor of Cdk4 and Cdk6, which are important regulators of interphase. U2-OS cells, which are deficient in p16, should be particularly sensitive to overexpression of this protein [34]. p21 (Cip1/Waf1) and p27 (Kip1) are members of the general Cip/Kip family of proteins, which promote assembly and thus activation of the cyclin D-Cdk4,6 complexes that control interphase, but inhibit the downstream cyclin-Cdk2 complexes that control the G1 to S transition and early S phase. All three of these node proteins are interdependent, as increases in cyclin D activates Cdk4,6 and sequesters Cip/Kip from downstream cyclin-Cdk2, which also stimulates division. Meanwhile, increased levels of p16 disassembles cyclin D-Cdk4,6, releasing Cip/Kip to bind cyclin-Cdk2, thereby arresting cells in G0/1 [38].

The screening assay also provided some unexpected results. For example, the overexpression of pRb and mutant forms of pRb, such as pRb(ΔCDK)HA and pRB(T821, 826A), which remove regulation by Cdk and inhibitory phosphorylation sites, respectively, [34, 39] did not lead to appreciable arrest in G0/1 (Fig. 1b). These results are surprising as pRb is an important inhibitor of the E2F-dependent gene expression cascade that triggers the transition of G1 to S, and heterologous expression of pRB(ΔCDK)HA was previously shown to significantly arrest U2-OS cells in G0/1 [34]. Taken together, our results highlight that functional screens within the desired cell system are important for identifying effective regulatory nodes of complex networks controlling sophisticated cellular phenotypes.

### Identifying key regulatory nodes that arrest cells in G2/M

Based on our success in identifying key regulatory nodes for inhibiting progression of the G0/1 to S transition, we applied a similar screening strategy to identify key regulatory nodes for inhibiting progression of the G2/M to G0/1 transition. A panel of putative regulatory node proteins for the G2/M to G0/1 transition were identified based on a review of the literature for most likely candidates (Additional file 1: Table S1). The cDNAs for these candidate proteins were overexpressed from a mammalian expression plasmid in U2-OS cells by transiently co-transfecting 3–3.75 μg of the individual plasmids encoding the expression of these candidate proteins with 1.13-1.5 μg of a plasmid encoding a GFP reporter for use as a transfection marker for a total of 4.5 μg transfected DNA per sample. The distribution of cells across the cell cycle phases for each sample was quantified via DNA staining using similar assay conditions as previously described.

The data from the screening assay indicate a single regulatory node for inducing a G2/M arrested cell state in U2-OS cells - cyclinB1mut (CCNB1m) (~44 % in G2/M) (Fig. 1c). The negative control (i.e., cells harboring a plasmid encoding DsRed expression) results in ~26 % of cells in G2/M under the same conditions. Thus, overexpression of the key node cyclinB1mut (CCNB1m) results in ~70 % more cells in G2/M. For the G2/M transition to G0/1, a single protein in a large network, which contains at least 131 proteins annotated as “G2/M transition of mitotic cell cycle” by the Gene Ontology project (or ~0.8 % of the network), was able to significantly increase the percentage of cells in G2/M (AmiGO 2.2.0) [37]. It is of note that only a mutant form (L45A, R42A) of cyclin B1 [40], which is less susceptible to degradation, had an effect on cell cycle progression, while wild-type cyclin B1 and the closely related cyclin B2 had no significant effect. It is of note that these conclusions are specific to U2-OS cells at three days after transfection and not necessarily generalizable to other cell lines or other time points in a transient transfection assay.

The identification of the cyclinB1mut as a key regulator of the G2/M to G0/1 transition is supported by its role in the cell cycle network (Fig. 1a). The cyclin B-Cdk1 complex activates the anaphase-promoting complex (APC) in early mitosis and is subsequently destroyed by APC in late mitosis. Destruction of the cyclin B-Cdk1 complex is necessary to transition out of mitosis, and thus a non-degradable cyclin B would be expected to induce arrest in M phase. Our observation that overexpression of wild-type cyclin B does not induce measurable arrest in G2/M (Fig. 1c) indicates that the amount of overexpression achieved under the assay conditions is insufficient to overwhelm the capability of APC. It was unexpected that the overexpression of several of the other candidate proteins did not lead to arrest in G2/M. For example, Emi1 was not identified as a key regulatory node of G2/M in this screen (Fig. 1c). Emi1 inhibits Cdc20, and activated APC(Cdc20) is necessary for the metaphase to anaphase transition in mitosis. Thus, overexpression of Emi1 was expected to result in an increased percentage of cells in S and G2/M [35].

Taken together, the results of our screening assays indicate that it was possible to identify key regulatory protein nodes whose overexpression produces modulation of the population level distribution of cells in G0/1 and G2/M despite the extensive network of proteins involved in controlling cell cycle phases.

### Optimizing arrest phenotypes by tuning expression of cell cycle regulatory nodes

We set out to determine methods for optimizing the amount of cell cycle arrest that can be achieved. Previous studies have demonstrated that simultaneously expressing multiple regulatory nodes produced increased arrest compared to individual expression of the nodes [34]. Thus, we examined the effects of combined overexpression of multiple nodes. In addition, genome-wide analyses of cell cycle phenotypes have reported large differences in the regulators identified across cell types [41, 42]. Thus, we examined the importance of cell type on the effectiveness of identified regulators. Previous work has shown that the arrangement of multiple expression cassettes within and across plasmids can impact expression [43]. In particular, the placement of two expression cassettes in tandem on the same plasmid can dampen expression of one or both genes [44–47], while the placement of two expression cassettes across two different plasmids can result in heterogeneity between cells [43]. Thus, we examined whether key regulatory node expression is more effective in cell cycle arrest when co-expressed with a GFP reporter as tandem expression cassettes on the same plasmid or separate cassettes across two plasmids.

**Fig. 2:**
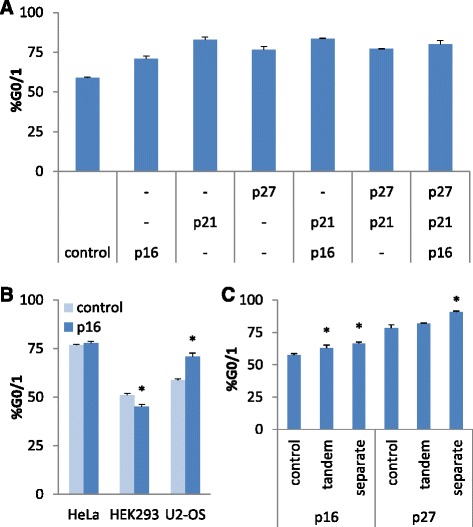
Impact of experimental parameters on the activity of regulatory nodes for cell cycle control. **a** Impact of combinatorial expression of key regulatory nodes on cell cycle arrest in G0/1. U2-OS cells were transiently co-transfected with plasmids encoding overexpression of combinations of one, two, and three key node proteins. The resulting arrest in G0/1 of the cell population was assayed via DNA staining and flow cytometry. **b** Impact of p16 on cell cycle arrest in G0/1 of different cell lines. HeLa, HEK293, or U2-OS cells were transiently transfected with a plasmid encoding p16 or a control and the percentage of the cell population in G0/1 was measured. **c** Impact of the arrangement of expression cassettes encoding regulatory nodes on cell cycle arrest in G0/1. U2-OS cells were transiently transfected with a single plasmid containing two expression cassettes, encoding a transfection marker and a regulatory node (p16 or p27) in tandem, or with two plasmids separately encoding the transfection marker and the regulatory node. The resulting arrest in G0/1 of the cell population was assayed. *, *p* < 0.05. Error bars represent standard deviation across biological triplicates

We identified multiple key nodes that inhibit the G0/1 to S transition, and thus we tested whether overexpressing these nodes in combination would improve arrest in G0/1. The three most promising candidates for arrest in G0/1 - p16, p21, and p27 - all regulate cell cycle through Cdk4 and Cdk6, and therefore would be expected to function synergistically. Plasmids encoding each of the proteins separately were transiently transfected singly or in combination at equal ratios into U2-OS cells and the percentage of cells in each stage of the cell cycle was measured as described previously. Under the assay conditions, combinations of the G0/1 regulatory nodes did not improve the amount of G0/1 arrest relative to the level of arrest observed from overexpression of a single key regulator (Fig. 2a). This result was unexpected as RNAi knockdown of Cdk4 and Cdk6 together results in more cells arrested in G0/1 compared to cell populations in which each Cdk was knocked down individually (Additional file 1: Figure S4). However, the mechanism of inactivation of Cdk4 and Cdk6 by p16, p21, and p27 is different, and perhaps less complete when compared to direct silencing via RNAi.

In order to investigate the impact of cell type on the ability of the identified regulatory nodes to arrest the cell cycle, we selected HeLa and HEK293 as representative commonly used cell lines and p16 as a representative key regulator of the G0/1 to S transition. Each cell line was transiently transfected with a plasmid encoding the expression of p16 or a negative control plasmid encoding the expression of a GFP reporter and cell cycle was measured after three days. The data shows that overexpression of p16 does not perturb the cell cycle relative to the negative control in HeLa cells, while overexpression of p16 decreases the percentage of cells in G0/1 slightly from 51 % in the control samples to 45 % in the experimental samples in HEK293 cells (Fig. 2b). U2-OS cells, but not HeLa or HEK293 cells, are expected to be especially sensitive to p16 overexpression because they are deficient in p16 [34]. Our results highlight that functional screens used to identify key regulatory nodes in the cell cycle network may identify candidates that are cell line dependent based on the underlying network in that cell line. Thus, conducting the screen in a cell line of interest is important for establishing an active system when engineering sophisticated cellular phenotypes.

We next investigated whether the expression of the key regulatory node protein is more effective in arresting cells in the cell cycle when co-expressed with a reporter protein as tandem expression cassettes on the same plasmid or as separate cassettes across two separate plasmids. Two representative regulators of G0/1, p16 and p27, were cloned into a mammalian expression plasmid such that a GFP reporter and the regulator expression cassettes were arranged in tandem. Cells were transiently transfected with the tandem cassette plasmid as described above and the level of cell cycle arrest compared to that from cells co-transfected with plasmids that separately encoded the two expression cassettes. The negative control consisted of a plasmid encoding DsRed expression, which is not expected to impact cell cycle arrest. The data shows that for both p16 and p27, the degree of arrest in G0/1 is increased when the expression cassettes are separated across plasmids relative to when they are arranged in tandem on a single plasmid (Fig. 2c). These results indicate that the particular arrangement of multiple expression cassettes has an important impact on regulation of the cell cycle, and under the assay conditions used in this study using two cassettes in tandem is detrimental. Thus, these results indicate that the optimal configuration to engineer switchable cell cycle controllers is to incorporate a single key regulatory node in U2-OS cells.

### Small molecule control of cell cycle arrest through ribozyme switches

**Fig. 3:**
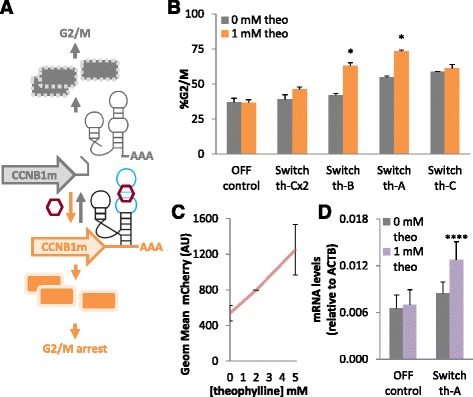
Small molecule-responsive ribozyme switches control arrest of cells in G2/M. **a** Schematic of the mechanism by which ribozyme switches mediate small molecule-dependent transition of cells into a G2/M arrest state, rather than the normal G2/M state, by regulating expression of CCNB1m. **b** A set of theophylline-responsive switches and a non-switch control (wild-type sTRSV hammerhead ribozyme; OFF control) were inserted in the 3′ UTR of CCNB1m, stably integrated into U2-OS T-Rex Flp-In cells, and tested with 0 or 1 mM theophylline (theo) for their ability to arrest cells in G2/M. **c** Characterization of gene-regulatory activity of switch th-A measured as reporter protein activity as a function of theophylline concentration. HEK293 cells were transiently transfected with a plasmid encoding switch th-A in the 3′ UTR of a mCherry reporter and induced with varying levels of theophylline. Mean fluorescence of the population was measured by flow cytometry. **d** Characterization of gene-regulatory activity of switch th-A measured as CCNB1m transcript levels in the presence and absence of theophylline. Cell lines harboring the stably integrated OFF control and switch th-A in the 3’UTR of the CCNB1m expression cassette were grown in 0 or 1 mM theophylline and CCNB1m transcript levels relative to that of a housekeeping gene (ACTB) were measured by qRT-PCR. *, *p* < 0.05, ****, *p* < 1E-4. Error bars represent standard deviation across biological triplicates for (**b**) and duplicates for (**c**) and (**d**)

Having identified key regulatory nodes for the arrest of human cells in G0/1 and G2/M, we next placed the expression of the identified regulatory proteins under the control of small molecule-responsive ribozyme switches. Ribozyme switches responsive to the small molecule theophylline were inserted into the 3′ UTR of the expression cassette for the regulatory node, where small molecule dependent cleavage modulates mRNA degradation and thus transcript levels of the regulatory node (Fig. 3a) [29]. The activities for a set of previously described theophylline-responsive switches (th-A, th-B, th-C) [31, 32] were characterized in the cell cycle control system. The switch set was chosen to exhibit a range of ligand-responsiveness as it was not known a priori which activity ranges would best match those of the identified regulatory nodes to allow effective control over the cell cycle. Based on the performance of these switches in yeast (Additional file 1: Table S2), switch th-C is expected to have the highest level of leakiness (i.e., basal expression in the absence of ligand) and the lowest level of activation upon addition of ligand, while switch th-A is expected to have the lowest level of leakiness and the highest activation. Introducing a second copy of switch th-C (th-Cx2) is expected to decrease the leakiness and increase the activation compared to the single switch th-C as described previously [32].

The ribozyme switches and non-switch negative controls (wild-type sTRSV hammerhead ribozyme, OFF control; mutated sTRSV hammerhead ribozyme, ON control) were cloned into the expression cassette for the G2/M regulatory node CCNB1m. The construct was placed under the control of a CMVTetO2 promoter and encoded on a plasmid backbone that allowed site-specific stable integration into U2-OS T-Rex Flp-In cells via the Flp-In system. We initially tested the ribozyme switches for control over G2/M arrest, because our earlier results indicated that a higher dynamic range is possible from a G2/M control system since a higher fraction of the population is available to be arrested relative to G0/1. The assay was performed on cell lines that expressed the cell cycle control construct through a single site-specific integration such that small molecule induced changes in cell cycle could be more precisely measured. The expression cassette for the regulatory node was placed under Tet-inducible control to allow stable cell line creation without interference from the effects of the cell cycle control system (i.e., in the absence of doxycycline), which would bias selection against the desired cell line since cells with the activated control system would be expected to grow more slowly. To characterize performance of the integrated constructs, the resulting cell lines harboring the cell cycle controllers were induced with doxycycline and either 0 or 1 mM theophylline (theo) for three days. Cell lines were then assayed for the percentage of cells in the different phases of the cell cycle by flow cytometry analysis as previously described.

The results for the non-switch controls demonstrated the expected trends (Additional file 1: Figure S5) and the different switches exhibited activities in line with the yeast activity data (Additional file 1: Table S2). Specifically, th-C was the most leaky switch tested, with 59 % of the cell population in G2/M in the absence of theophylline compared to the OFF control for which only 37 % of the cell population was in G2/M (Fig. 3b). While switch th-C exhibits ligand responsiveness in yeast and in mammalian cells as measured by fluorescent reporters (Additional file 1: Table S2), it is ineffective in the context of the cell cycle control system and does not increase the percentage of cells in G2/M with the addition of theophylline. Adding a second copy of switch th-C (th-C2x) lowers the amount of leakiness to only 39 % of the cell population in G2/M and restores ligand responsiveness to the control system, albeit by a modest amount (i.e., 46 % of the cell population in G2/M in the presence of theophylline). Switch th-B exhibited less leakiness and a better activation ratio than switch th-A in the context of the cell cycle control system, which was different than their relative activities observed in yeast (Additional file 1: Table S2). Specifically, cells harboring switch th-B have 42 % of the cell population in G2/M in the absence of theophylline, compared with 55 % from cells harboring switch th-A. However, after induction with theophylline, the cell population in G2/M increases 1.5-fold in cells harboring switch th-B, compared with 1.3-fold in cells harboring switch th-A.

We selected the theophylline switch (th-A), which arrested the largest percentage of the cell population in the presence of theophylline (up to 73 %), and verified the underlying mechanism via changes in the levels of the target protein and transcript as a function of theophylline. The gene-regulatory activity of switch th-A was verified by characterizing its activity in the context of an expression cassette for an mCherry reporter. The switch was cloned into the 3′ UTR of an mCherry expression cassette, and the plasmid encoding this construct was transiently transfected into HEK293 cells. The cells were induced with varying levels of theophylline, and fluorescence was measured by flow cytometry after three days of growth. The results indicate that the presence of theophylline increases the amount of reporter protein expression in a concentration dependent manner (Fig. 3c). We further verified that the transcript levels of CCNB1m increased with addition of theophylline to the cells. The cell lines harboring the stably integrated OFF control or switch th-A were induced with doxycycline and either 0 or 1 mM theophylline for three days. Total RNA was extracted from the samples and the levels of the CCNB1m transcript relative to those of a housekeeping gene (ACTB) were measured by quantitative real-time PCR (qRT-PCR). The results indicate that for a control system harboring the th-A switch, CCNB1m mRNA levels increase by 1.5-fold upon addition of theophylline, whereas those from a control system harboring the OFF control do not change upon the addition of theophylline (Fig. 3d). Taken together, the data support that the ribozyme switch-based cell cycle control system functions through specific modulation of the levels of the key regulatory node in response to the cognate ligand.

### Inducible and dynamic control of multiple phases of the cell cycle through ribozyme switches

**Fig. 4:**
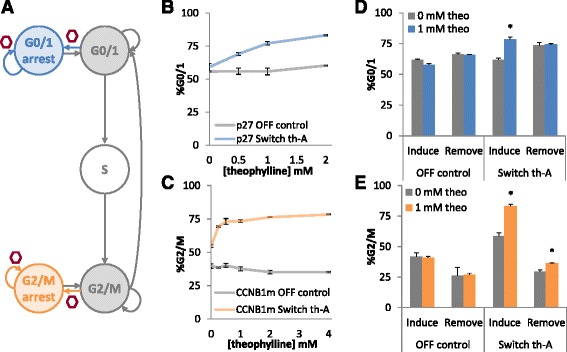
Small molecule responsive ribozyme switches for titratable and dynamic control over cell cycle. **a** Schematic of the progression through the phases of cell cycle (grey and white) with the introduction of the synthetic G0/1 cell cycle control system (blue) and synthetic G2/M cell cycle control system (orange). **b** Theophylline responsiveness of the G0/1 cell cycle control system (integrated p27-switch controller). Cell lines harboring the control system or a negative control were induced with a range of theophylline (theo) concentrations and assayed for the percentage of cells in G0/1 via DNA staining and flow cytometry. **c** Theophylline responsiveness of the G2/M cell cycle control system (integrated CCNB1m-switch controller). Cell lines harboring the control system or a negative control were induced with a range of theophylline (theo) concentrations and assayed for the percentage of cells in G2/M via DNA staining and flow cytometry. **d** The response of the G0/1 cell cycle control system to changes in theophylline concentration over time. Cell lines harboring the G0/1 control system (switch th-A) or a negative control (OFF control) were induced with 0 or 1 mM theophylline. After 3 days, samples were assayed for arrest in G0/1 by flow cytometry (induce) and re-seeded with 0 mM theophylline. After an additional 3 days, samples were assayed for G0/1 arrest (remove). **e** The response of the G2/M cell cycle control system to changes in theophylline concentration over time. Cell lines harboring the G2/M control system (switch th-A) or a negative control (OFF control) were induced with 0 or 1 mM theophylline. After 3 days, samples were assayed for arrest in G2/M by flow cytometry (induce) and re-seeded with 0 mM theophylline. After an additional 3 days, samples were assayed for G2/M arrest (remove). *, *p* < 0.05. Error bars represent standard deviation across triplicates

Having shown that small molecule-responsive ribozyme switches can be applied to control progression of human cells through the G2/M to G0/1 transition, we next examined whether a similar strategy could be used to control progression through the G0/1 to S transition (Fig. 4a). We selected p27 as the regulatory node for G0/1 arrest as it was the most effective of the nodes identified for inducing cell cycle arrest in G0/1 across multiple experiments. We further examined the ligand responsiveness and dynamics of cell cycle controllers based on ribozyme switches with these cell lines.

To build a cell cycle controller for G0/1, the theophylline-responsive switch th-A and the non-switch controls (wild-type sTRSV hammerhead ribozyme, OFF control; mutated sTRSV hammerhead ribozyme, ON control) were cloned into the expression cassette for the G0/1 regulatory node p27. The construct was placed under the control of a CMVTetO2 promoter and encoded on a plasmid backbone that allowed site-specific stable integration into U2-OS T-Rex Flp-In cells via the Flp-In system. The resulting U2-OS cell lines harboring the cell cycle controller for G0/1 (integrated p27-switch controller), the cell cycle controller for G2/M (integrated CCNB1m-switch controller), or the respective controls were induced with doxycycline and a range of theophylline concentrations for three days. Cell lines were then assayed for the percentage of cells in the different phases of the cell cycle through flow cytometry analysis as previously described.

The synthetic G0/1 cell cycle control system responds to varying theophylline concentrations by increasing the percentage of cells in G0/1. Specifically, the G0/1 control system begins to respond to inducer concentrations as low as 0.5 mM, exhibits a maximal switching range at around 1 mM, and begins to show some non-specific effects (i.e., OFF control statistically deviates from no induction sample) at 2 mM theophylline (Fig. 4b). When induced with 1 mM theophylline, 77 % of the cells are in G0/1 compared to 59 % when uninduced, which represents a significant increase (p-value < 0.05). The percentage of cells arrested in G0/1 by the control system is close to the expected maximum performance of this system as determined by the percent of cells in G0/1 in the ON control system (Additional file 1: Figure S6). Similar to the initial screen with transient expression of this regulatory node, the integrated p27-switch control system controlled up to 44 % of the cells that were previously escaping G0/1 arrest. There is a small amount of leakage in the device as the uninduced switch cell line exhibits slightly more cells in G0/1 (only 3 % more) compared to the OFF control, especially when compared to the cell cycle controllers for G2/M.

While the synthetic G2/M cell cycle control system similarly responds to varying theophylline concentrations there are quantitative differences in the response properties of the two control systems. Specifically, the synthetic G2/M cell cycle control system also exhibits a maximal switching range at around 1 mM theophylline and non-specific effects by 2 mM theophylline (Fig. 4c). At the maximal range of the control system, 73 % of cells are in G2/M compared to 55 % in the absence of theophylline. Thus, the integrated CCNB1m-switch device controls 55 % of the cells that were previously escaping G2/M arrest. This is substantially more than the control of 24 % of the previously escaped cells from transient expression of CCNB1m, which may be explained by fusion tags coupled to the CCNB1m coding region within the transient expression construct that were removed in the integration construct. The G2/M cell cycle control system exhibits a higher leakiness (~15 %) compared to the OFF control, which has ~40 % of cells in G2/M. Thus, the G2/M control system is 5 times more leaky than the G0/1 control system. In addition, the G2/M control system exhibits a greater sensitivity to the inducing ligand; near full induction of the system is observed at 0.25 mM theophylline. The observed quantitative differences in the response properties between the cell cycle controllers for G0/1 and G2/M are likely due to the differences in the mechanism of the regulatory nodes.

We next examined the reversibility of the genetic cell cycle control systems, or their ability to respond to changes in the concentration of the small molecule inducer. The cell lines harboring the cell cycle controller for G0/1 (integrated p27-switch controller), the cell cycle controller for G2/M (integrated CCNB1m-switch controller), and the respective controls were induced with doxycycline and 0 or 1 mM theophylline with six samples per condition. After three days of growth, three samples from each condition were assayed for the percentage of cells in the different phases of the cell cycle and three samples were re-seeded in fresh media with doxycycline and no theophylline. After another three days of growth, the latter samples were assayed for the percentage of cells in the different phases of the cell cycle through flow cytometry analysis as previously described.

Cells harboring the cell cycle controller for G0/1 initially displayed an increased percentage of cells in G0/1 (16 % more) and subsequently return to levels of G0/1 identical to samples that were uninduced throughout the entire experiment (Fig. 4d). Cells harboring the cell cycle controller for G2/M also initially showed an increased percentage of cells in G2/M, with 83 % of the total cells arrested before reverting to a much lower level of 36 %. However, the percentage of cells in G2/M revert to levels slightly higher than those from samples that were uninduced throughout the entire experiment, which were at 29 % (Fig. 4e). These results indicate that the effects from the genetic cell cycle control systems are generally reversible within three days of removal of the inducing molecule from the cells.

Overall, the results demonstrate that ribozyme switches can modularly and reversibly arrest human cells in different phases of the cell cycle by linking them to the expression of key regulators of the cell cycle.

### Reliability of ribozyme switch-based cell cycle control systems

One important property of synthetic biological circuits is their reliability over time, particularly as it relates to complex phenotypes. Thus, we examined the performance of the cell cycle control systems over time and across experiments by leveraging the stable cell lines generated through the course of our experiments.

**Fig. 5:**
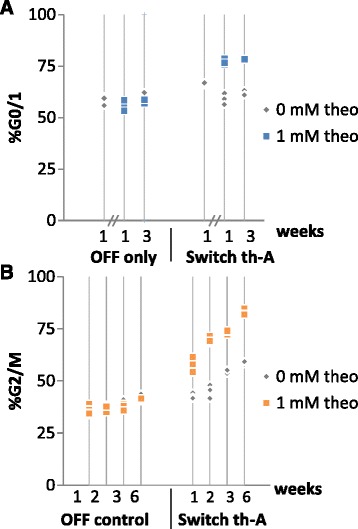
Reliability of cell cycle control systems over time. **a** Performance of the cell cycle controller for G0/1 (integrated p27-switch controller) over time. Cells harboring the G0/1 cell cycle controller (p27 switch th-A) or a negative control (p27 OFF control) were induced with 0 or 1 mM theophylline (theo) and the percentage of the cell population in G0/1 was measured via DNA staining and flow cytometry after creation of the cell lines (left of hash marks) and at 1 and 3 weeks after cell lines were frozen and revived (right of hash marks). **b** Performance of the cell cycle controller for G2/M (integrated CCNB1m-switch controller) over time. Cells harboring the G2/M cell cycle controller (CCNB1m switch th-A) or a negative control (CCNB1m OFF control) were induced with 0 or 1 mM theophylline and the percentage of the cell population in G2/M was measured via DNA staining and flow cytometry at 1, 2, 3 and 6 weeks after creation of the cell lines

Cell cycle measurements performed on the cell lines harboring the cell cycle controller for G0/1 (integrated p27-switch controller), the cell cycle controller for G2/M (integrated CCNB1m-switch controller), and the respective controls three days after induction with doxycycline and either 0 mM or 1 mM theophylline from several independent experiments were compared. The aggregated results show that the cell cycle controller for G0/1 is highly reliable, with no significant deviation when comparing performance before and after a freeze-thaw cycle nor when comparing performance over 3 weeks (Fig. 5a). In contrast, aggregated results for the cell cycle controller for G2/M show a significant increase in the measured arrest in G2/M over time, with the difference in the percentage of cells measured to be in G2/M drifting by at least 4 % (for cell lines harboring the OFF control) and up to 20 % (for cell lines harboring switch th-A) (Fig. 5b). This is especially prominent in the CCNB1m-th-A cell line which change from ~60 % cells in G2/M during week one to ~80 % cells in G2/M during week six in the presence of theophylline and from ~40 % to 60 % in the absence of theophylline. The observed drift in percent of cells in G2/M over time from this cell line may be due to the overexpression of CCNB1m, which contains mutations that protect it from the naturally regulated degradation pathway and thus is quite long lived and likely accumulating in the cell line over time, ultimately leading to increased susceptibility to arrest in G2/M. Taken together, the results indicate that the specific choice of key regulatory node that is implemented in these genetic control systems can impact the reliability of control system activity over time.

## Conclusion

Despite the complexity and scale of the endogenous gene networks that control human cell cycle transitions, it is possible to identify key regulatory nodes that induce population level increases in the percentage of cells in a population that are in the G0/1 phase or the G2/M phase of the cell cycle. Small molecule-responsive ribozyme switches can be modularly applied to control arrest of cells in the G0/1 or the G2/M phases of the cell cycle by controlling the identified key regulators of the cell cycle. The resulting synthetic cell cycle control systems dynamically respond to changing levels of the cognate small molecule inducer. Finally, ribozyme-based controllers of the cell cycle can be highly reliable, reproducibly performing with no significant deviation over the course of several weeks.

Compared to currently available small molecule inhibitors of the cell cycle or nutrient deprivation methods [20], a switchable ribozyme gene expression control platform for inducible cell cycle arrest has several advantages. For example, the switches can be programmed to respond to both exogenous small molecule inputs as well as endogenous protein inputs [31, 48] by swapping out the aptamer region of the switch, whereas current chemical methods are limited to control by exogenous inputs only. Therefore, proteins serve as generalizable biological connectors between genetic switches and devices, as well as between the native and synthetic programs [49–51]. In addition, the rate of development of new small molecule cell cycle inhibitors is slowing [52]. In contrast, while this first generation of ribozyme cell cycle controller is less effective (~80 % cells are arrested in the desired phase), rapid development of new generations is enabled by the availability of high-throughput approaches for screening target proteins as well as new switches [30, 31, 48].

Ribozyme switches are an effective, inducible, and reversible method of controlling arrest of human cells in both G0/1 and G2/M. To our knowledge, this is the first time synthetic RNA devices have been used to control the mammalian cell cycle. Furthermore, these cell cycle controllers can be readily adapted to arrest cells in other phases of the cell cycle, in response to a variety of different inputs, and to be part of larger synthetic programs. Thus, RNA devices are a promising synthetic biology tool that enables the engineering of sophisticated multi-output cellular programs in higher organisms.

## Methods

### Plasmid construction

Plasmids were constructed using standard molecular biology techniques [53]. Oligonucleotides were synthesized by Integrated DNA Technologies or the Stanford Protein and Nucleic Acid Facility (PAN, Stanford, CA) and constructs were sequence verified (Laragen, Inc., Culver City, CA or Elim Biopharmaceuticals, Inc., Hayward, CA). Cloning enzymes, including restriction enzymes and T4 DNA ligase, were obtained from New England Biolabs (Ipswich, MA), and DNA polymerases were obtained from Stratagene (Agilent Technologies, Santa Clara, CA). Plasmids were prepared from *Escherichia coli* using Econospin columns (Epoch Life Science, Missouri City, TX) or PureYield plasmid miniprep system (Promega Corporation, Madison, WI) according to manufacturer’s instructions. For a detailed description of plasmid construction methods see Additional file 1: Text S1 and Figure S7. Lists of plasmids and ribozyme switch sequences are provided in Additional file 1: Tables S5 and S6.

### Mammalian cell culture

U2-OS cells (a generous gift from the Katrin Chua Laboratory, Stanford, CA), HeLa cells (a generous gift from the James Chen Laboratory, Stanford, CA), and HEK293 were cultured in D-MEM media with 10 % FBS and passaged regularly. Parental U2-OS T-Rex Flp-In cells (a generous gift from the Pamela Silver Laboratory [14]) were maintained in DMEM supplemented with 10 % FBS, 0.1 mg/ml zeocin (Life Technologies, Carlsbad, CA), and 2.5 μg/ml blasticidin (Life Technologies). All cells were grown at 37 °C, 5 % CO_2_, and 80 % humidity. Stable transfection of U2-OS T-REx FlpIn cell lines was performed using the Flp-In recombinase system (Life Technologies) according to the manufacturer’s instructions to generate isogenic stable cell lines. Stable integrants were selected using 0.2 mg/ml hygromycin B (Life Technologies), whereas stable cell lines were maintained in 0.1 mg/ml hygromycin B and 2.5 μg/ml blasticidin. For a list of cell lines, see Additional file 1: Table S3.

### qRT-PCR assays

Stable U2-OS cell lines of interest were seeded at 0.02 × 10^6 cells/ml in 6 cm plates with 25 ng/ml doxycycline and 0 or 1 mM theophylline (Sigma-Aldrich, St. Louis, MO) in duplicate. 72 h after seeding, supernatant along with trypsinized cells were collected by spinning at 300 g for 5 min. Samples were washed once with PBS in 1.5 ml microfuge tubes and used PBS was aspirated. Cell pellets were flash frozen in liquid nitrogen and stored at −80 °C.

RNA extraction was performed using GenElute™ Mammalian Total RNA Miniprep Kit (Sigma-Aldrich) according to the manufacturer’s instructions. Reverse transcription with primer 26_ACTB_REV or B1m-4_rv (Additional file 1: Table S4) was performed using SuperScript III reverse transcriptase (Life Technologies) according the manufacturer’s instructions using at least 200 ng of total RNA. qPCR was performed using EvaGreen master mix (Biotium, Hayward, CA) using 15 ng of template and 0.5 μM each of primers 25_ACTB_FWD and 26_ACTB_REV to measure the housekeeping control (ACTB) and primers B1m-4_fw and B1m-4_rv to measure CCNB1m transcript levels (Additional file 1: Table S4) according to the manufacturer’s instructions on a Bio-Rad iCycler with a cycle of 95 °C (15 s), 55 °C (15 s), and 72 °C (30 s) run at least 45 cycles. Relative expression was calculated by the ΔC_T_ method according to the manufacturer’s manual.

### Fluorescence reporter assays

HEK293 cells were seeded at 0.16 × 10^6 cells/ml in 24-well plates. 24 h after seeding, cells were transfected with FuGENE HD (Promega) according the manufacturer’s instructions using 500 ng total plasmid and induced with 0, 2, or 5 mM theophylline. 72 h after transfection, at least 5000 cells were collected and run on a MACSQuant VYB (Miltenyi Biotec, Bergisch Gladbach, Germany) and mCherry was excited by a 561 nm laser and detected by a 615/20 band-pass filter. The data was gated for live cells using side scatter vs. forward scatter and the geometric mean fluorescence of the mCherry signal was determined using FlowJo software (Tree Star, Ashland, OR). The average and standard deviation across biological duplicates are plotted.

### Stable cell line cell cycle assays

Stable U2-OS cell lines of interest were seeded at 0.01–0.02 × 10^6 cells/ml in 6 cm plates with 25 ng/ml doxycycline and 0 to 4 mM theophylline (Sigma). 72 h after seeding, DNA content was measured following propidium iodide (PI) staining according to previously described protocols modified to use 100 % methanol for >10 min instead of 70 % ethanol and a final concentration of 20 μg/ml PI rather than 10 μg/ml [54]. At least 10,000 cells from samples were run on a MACSQuant VYB (Miltenyi Biotec) and PI staining was excited by a 561 nm laser and detected by a 615/20 band-pass filter output linear. The data was gated to exclude debris using side scatter vs. forward scatter, then for singlets using the height of forward scatter vs. the area of the forward scatter signal. Next, histograms of cell frequency vs. DNA content were gated manually for cells in G0/1, S, and G2/M phase using FlowJo software (Tree Star, Ashland, OR) with the leftmost peak representing 1n chromosome staining, or cells in G0/1, the rightmost peak representing 2n chromosome staining, or cells in G2/M, and intermediate staining representing cells in S, as described in Pozarowski et al. (Additional file 1: Figure S1, S2).

For reversibility experiments, 72 h after initial seeding, the cell cycle assay was performed on samples as described above and samples were re-seeded to 0.01–0.02 × 10^6 cells/ml in 6 cm dishes in fresh media with 25 ng/ml doxycycline and no theophylline. After an additional 72 h of growth, DNA content was measured with PI staining and an analysis of the percentage of cells in G0/1, S, and G2/M was performed as described above.

### Transient cell cycle assays

For transient assays, U2-OS cells were seeded at 0.02 × 10^6 cells/ml in 6 cm plates. 24 h after seeding, cells were transfected with FuGENE HD (Promega) according to the manufacturer’s instructions using 4.5 μg total plasmid. During our initial protocol optimization, the total amount of DNA transfected (i.e., transfection marker plasmid and regulatory protein plasmid) was determined by the amount that produced the best transfection efficiency in U2-OS cells using FuGENE HD in 6-well plates while maintaining a low enough toxicity such that >10,000 GFP positive cells per sample could be quantified in the flow cytometry assay. At this upper limit of total transfected DNA (i.e., 4.5 μg) the ratio of the amount of transfection marker plasmid relative to the amount of plasmid encoding the protein of interest was then optimized to maximize the amount of protein of interest plasmid that was added while preserving high enough levels of GFP signal at the time of assaying the cells to allow quantification of >10,000 GFP positive cells per sample. For 2 plasmid co-transfections, 1.125–1.5 μg of pCS2622 and 3–3.375 μg of the second plasmid were used. For greater than 2 plasmid co-transfections, equal mass of each plasmid was added.

Seventy two hours after transfection, DNA staining was performed as described above modified to replace methanol permeabilization. Specifically, cells were fixed using 0.5 % formaldehyde for 20 min at 4 °C, washed once with PBS, then permeabilized with 70 % ethanol for at least 2 h. During the initial protocol optimization, it was determined that 72 h after transfection was a reasonable time to assay for cell cycle changes because at this time point the cells had sufficiently recovered from transfection, the density of cells in the dishes was high enough that >10,000 GFP positive cells per sample could be reliably quantified, and the cell density was not so high that contact inhibition of growth would begin to mask cell cycle arrest induced by the overexpression of the potential regulatory node proteins being screened. In addition to the data processing described above, after gating for singlets, GFP (excited by a 488 nm laser and detected by a 525/50 band-pass filter) signal is used to gate for transfected cells before analysis of DNA staining in the GFP positive subpopulation (Additional file 1: Figure S1). Note that the GFP reporter was membrane-tagged to ensure GFP signal is preserved after fixation step [55].

## Additional file

Additional file 1: Text S1. Plasmid construction. **Figure S1.** Overview of DNA staining and cell cycle quantification experiments. **Figure S2.** Representative histograms of cell count vs. DNA staining. **Figure S3.** Double thymidine block. **Figure S4.** siRNA knockdown of CDK4, CDK6, or both CDK4 and CDK6 in U2-OS cells. **Figure S5.** Ribozyme switch controls for arresting cells in G2/M. **Figure S6.** Ribozyme switch controls for arresting cells in G0/1. **Figure S7.** Plasmid maps. **Table S1.** Regulatory nodes tested in this study. **Table S2.** Ribozyme switch performance in yeast and HEK293. **Table S3.** Cell lines created in this study. **Table S4.** Primer sequences. **Table S5.** List of the plasmids used in this study. **Table S6.** Sequences of ribozyme controls and switches used in this study. (PDF 1609 kb)
