# Supplementary material for: Engineering dynamic cell cycle control with synthetic small molecule-responsive RNA devices
Source: J Biol Eng. 2015 Nov 20;9:21. doi: 10.1186/s13036-015-0019-7 (PMC4654890; doi:10.1186/s13036-015-0019-7)
Supplement: Additional file 1: Text S1. — Plasmid construction. Figure S1. Overview of DNA staining and cell cycle quantification experiments. Figure S2. Representative histograms of cell count vs. DNA staining. Figure S3. Double thymidine block. Figure S4. siRNA knockdown of CDK4, CDK6, or both CDK4 and CDK6 in U2-OS cells. Figure S5. Ribozyme switch controls for arresting cells in G2/M. Figure S6. Ribozyme switch controls for arresting cells in G0/1. Figure S7. Plasmid maps. Table S1. Regulatory nodes tested in this study. Table S2. Ribozyme switch performance in yeast and HEK293. Table S3. Cell lines created in this study. Table S4. Primer sequences. Table S5. List of the plasmids used in this study. Table S6. Sequences of ribozyme controls and switches used in this study. (PDF 1609 kb) [file 13036_2015_19_MOESM1_ESM.pdf]

Engineering dynamic cell cycle control with synthetic small-molecule responsive RNA devices

Wei KY and Smolke CD

SUPPLEMENTARY INFORMATION

Text S1. Plasmid construction

Figure S1. Overview of DNA staining and cell cycle quantification experiments

Figure S2. Representative histograms of cell count vs. DNA staining

Figure S3. Double thymidine block

Figure S4. siRNA knockdown of CDK4, CDK6, or both CDK4 and CDK6 in U2-OS cells

Figure S5. Ribozyme switch controls for arresting cells in G2/M

Figure S6. Ribozyme switch controls for arresting cells in G0/1

Figure S7. Plasmid maps

Table S1. Regulatory nodes tested in this study

Table S2. Ribozyme switch performance in yeast and HEK293

Table S3. Cell lines created in this study

Table S4: Primer sequences

Table S5. List of the plasmids used in this study

Table S6: Sequences of ribozyme controls and switches used in this study

References

Text S1. Plasmid construction

All primers used in this study are listed in Table S4. All plasmids used in this study are listed in Table S5. All cell lines created and used in this study are listed in Table S2. Brief details on the construction of various plasmids built for this study are provided below and plasmid maps are drawn in Fig S7.

The plasmid construct used as the transfection marker to screen potential cell cycle regulatory nodes (pCS2622; Fig S7) is built on a pcDNA3.1(+) backbone (Life Technologies) and encodes an expression cassette for a transfection marker and the regulatory node. First, a fragment encoding a bGH polyA tail and the CMV promoter was PCR amplified from pCS1036 [1] using primers BsrGI_EcoRI_bGHpA_fwd and ApaI_XbaI_XhoI_CMV_rev. This fragment was inserted into pCS408 (pcDNA3.1(+) with EGFP cloned into the KpnI and XhoI restriction sites [1]) via the BsrGI and ApaI restriction sites to create CMV-EGFP-bGHpA-CMV-bGHpA. Next, a fragment encoding an EGFP reporter with a Us9 localization tag (Us9-EGFP), which localized the reporter to the golgi and plasma membranes [2], was PCR amplified from pCS2052 (Addgene #18657) using primers Us9_KpnI_fwd and EGFP_EcoRI_rev. This fragment was inserted into the intermediate plasmid described above via the KpnI and EcoRI restriction sites. Finally, a fragment encoding the HSVTK polyA tail was PCR amplified from pCS2587 using primers HSVTK_EcoRI_fwd and HSVTK_EcoRV_rev. This fragment was inserted into the intermediate plasmid described above via the EcoRI and EcoRV restriction sites to create pCS2622.

Plasmid pCS2622-p16 (pCS3341), used as the tandem cassette for p16 in tuning expression of key regulators, was constructed by PCR amplifying p16 using primers p16_SalI_XbaI_fw and p16_ ApaI_rv and inserting into pCS2622 via XhoI and XbaI. Plasmid

pCMV p16 INK4A from which p16 was amplified was a gift from Bob Weinberg (Addgene plasmid # 10916).

Plasmid pCS2622-p27 (pCS3342), used as the tandem cassette for p27 in tuning expression of key regulators, was constructed by PCR amplifying p27 from using primers GB_2622_p27_fw and GB_2622_p27_rv and inserting into pCS2622 via KpnI and BamHI using Gibson assembly. Plasmid pCMV5 human p27 from which p27 was amplified was a gift from Joan Masague (Addgene plasmid #14049).

Plasmid pCS2441, the plasmid backbone for site-specific integration of constructs into cell lines, was built on the pcDNA5/FRT (Invitrogen) backbone such that the existing expression vector CMV-GOI-bGHpA is replaced by CMVTetO2-DsRed-bGHpA (Fig S7). Specifically, the CMV-d2EEGFP-bGHpA cassette in pCS2407 [3] was replaced by a multiple cloning site containing MfeI-NruI-NheI-AflII-HindII-AvrII-XmaI by annealing primers MCS_fwd and MCS_rev and ligating into the vector via MfeI/XmaI.

pCS2441-Wee1mut (pCS3246), used in screening potential regulatory nodes, was constructed by PCR amplifying Wee1mut using primers GB_2441_Wee1mut_fw and GB_2441_Wee1mut_rw and inserting into pCS2441 via BamHI and KpnI, replacing DsRed. Wee1mut sequence was ordered from Gen9 (Cambridge, MA).

pCS2441-syn21p27 (pCS3249), the base plasmid for G0/1 cell cycle controllers, was constructed by PCR amplifying p27 from pCMV5 human p27 (a gift from Joan Massague (Addgene plasmid # 14049)) using primers GB_2441_syn21_fw and GB_2441_p27_rv and inserting into pCS2441 BamHI and ApaI, replacing DsRed. The OFF control (sTRSV) and switch th-A (theoRs2) were PCR amplified using primers RzXhoIAsiSI_fw and RzApaIPacI_rv and inserted into the restriction sites XhoI and ApaI to form pCS2441-syn21p27-sTRSV

(pCS3250) and pCS2441-syn21p27-theoRs2 (pCS3252), which are used to test small molecule control of cell cycle arrest in G0/1.

pCS2441-syn21CCNB1mut (pCS3256), the base plasmid for G2/M cell cycle controllers, was constructed by PCR amplifying CyclinB1(L45A,R42A) from pCyclinB1(L45A,R42A)-mCherry (a gift from Jonathon Pines (Addgene plasmid # 39852) using primers BamHI_XbaI_CCNB1_fw and ApaI_XhoI_CCNB1_rv and inserting into the relevant pCS2441-switch vector via BamHI and ApaI, replacing DsRed. The OFF control (sTRSV) and switches were PCR amplified using primers RzXhoIAsiSI_fw and RzApaIPacI_rv and inserted into the restriction sites XhoI and ApaI to form pCS2441-syn21CCNB1mut-sTRSV (pCS3257), pCS2441-syn21CCNB1mut-theoRs2 (pCS3261; CCNB1mut Switch th-A), pCS2441-syn21CCNB1mut-theoRs4 (pCS3262; CCNB1mut Switch th-B), pCS2441-syn21CCNB1mut-L2b9(1x) (pCS3259; CCNB1mut Switch th-C), and pCS2441-syn21CCNB1mut-L2b9(2x) (pCS3260; CCNB1mut Switch th-Cx2), which are used to test small molecule control of cell cycle arrest in G2/M.

pCS2786-Cas9-mCherry-theoRs2 (pCS3256), used to characterize switch th-A (theoRs2) activity, was constructed by PCR amplifying a DNA fragment encoding a P2A cleavable linker, mCherry, and switch th-A with primers GB_Cas9_P2A_fw and GB_postRZ_rv from an existing plasmid and inserting into pCS2786-Cas9 via XbaI using Gibson assembly.

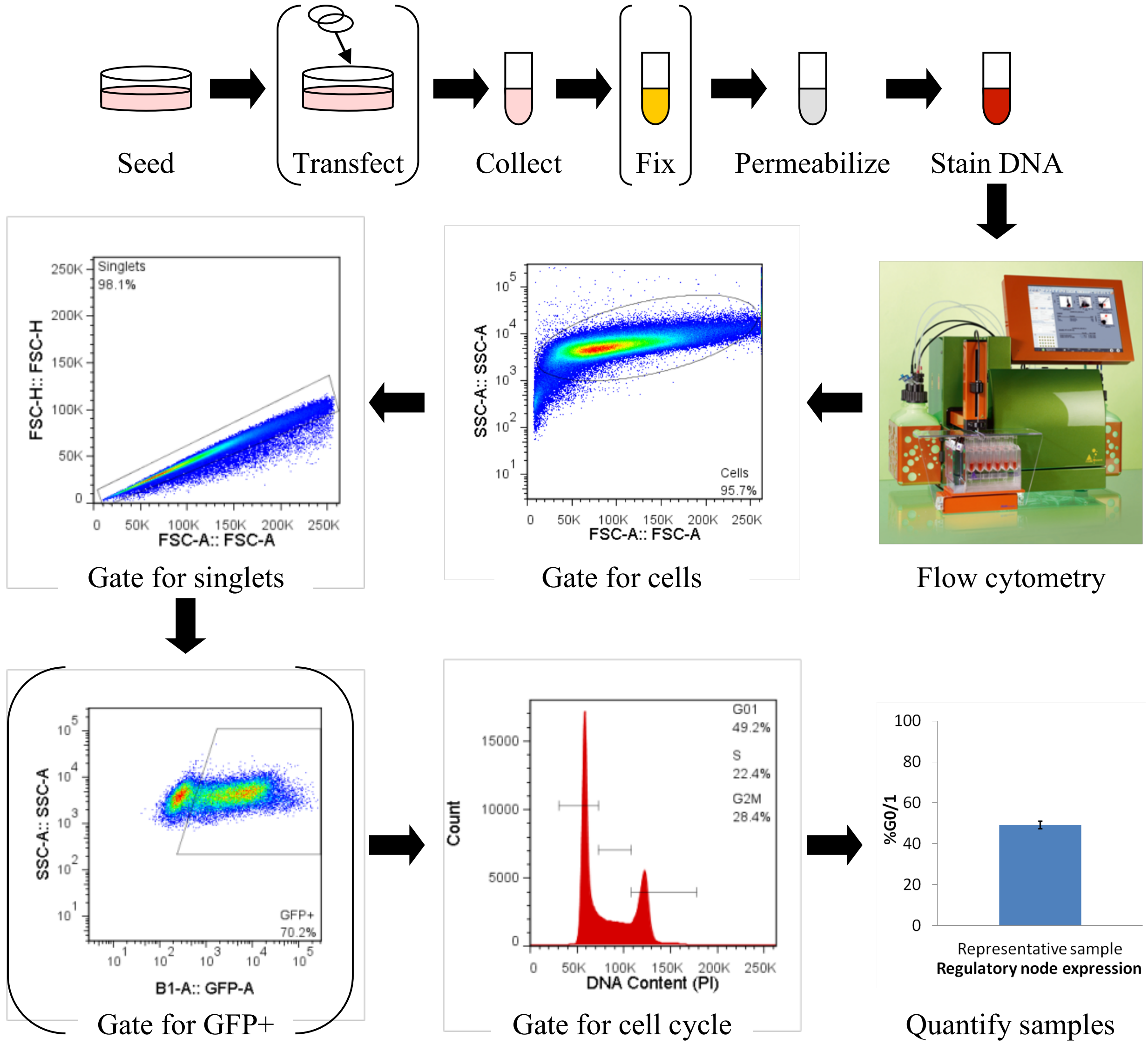

Figure S1: Overview of DNA staining and cell cycle quantification experiments. Generally, cells are seeded in dishes and grown for 3 days. Cells are then collected, permeabilized, and stained for DNA content and run on a flow cytometer. Each sample is analyzed by gating for cells using side scatter (SSC) vs. forward scatter (FSC) and for singlets using height of the forward scatter signal vs. the area of the forward scatter signal. The proportion of cells in G0/1 and G2/M can be quantified by gating on the amount of propidium iodide staining. The protocol with transient transfections has extra steps, shown in brackets including transfection, a fixation step, and gating on transfected cells using GFP.

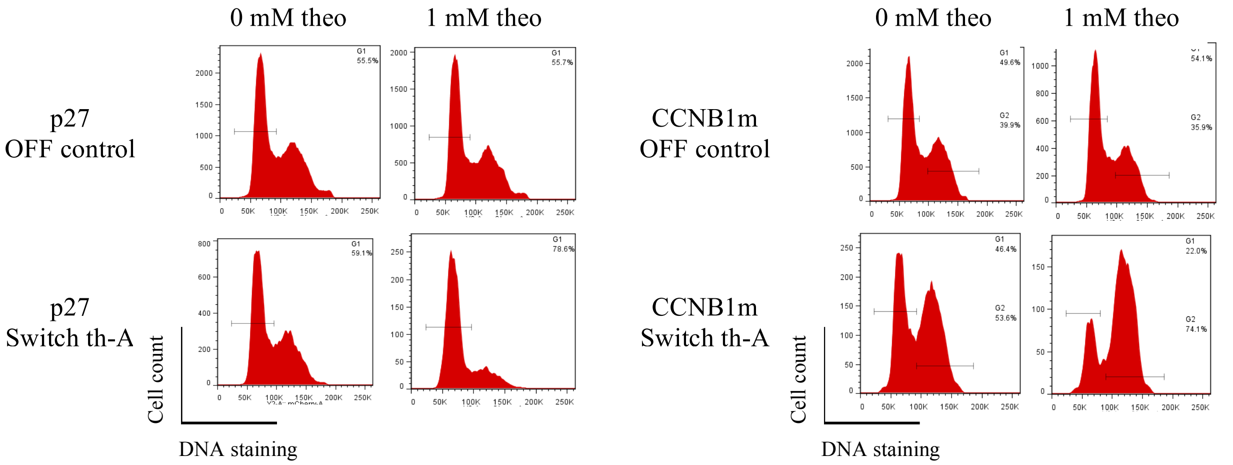

Figure S2: Representative histograms of cell count vs. DNA staining. Top row shows the OFF control, which does not change in the percentage of cells in any of the cell cycle phases between the addition 0 and 1 mM theophylline (theo). Bottom row shows the Switch th-A. For integrated p27-switch controllers (left), there is an increase in the percentage of cells in G0/1 (and correspondingly a decrease in G2/M) when 1 mM theo is added compared to when 0 mM theo is added. For integrated CCNB1m-switch controllers (right), there is an increase the percentage of cells in G2/M (and correspondingly decreases the percentage of cells in G0/1) when 1 mM theo is added compared to when 0 mM theo is added.

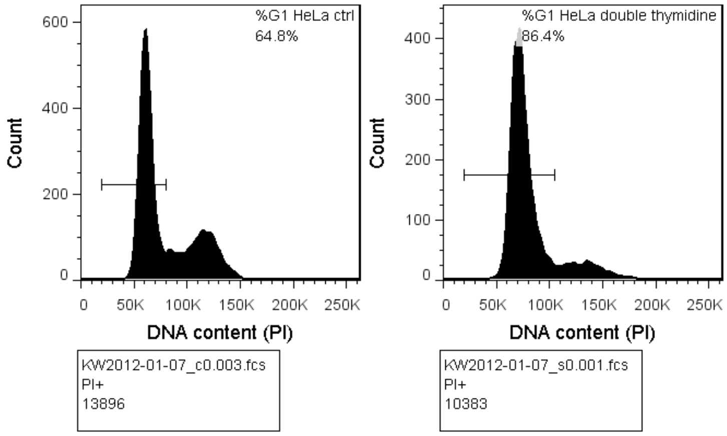

Figure S3: Double thymidine block treated (right graph) or control treated (left graph) HeLa cells. The percentage of cells in a population arrested in G0/1 was measured by DNA staining and flow cytometry.

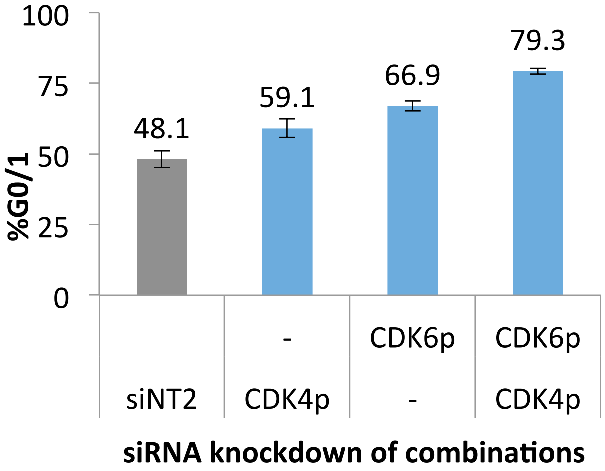

Figure S4: siRNA knockdown of CDK4, CDK6, or both CDK4 and CDK6 in U2-OS cells. siNT2 is a control sequence. The percentage of cells in a population arrested in G0/1 was measured by DNA staining and flow cytometry. Error bars represent standard deviation of triplicates.

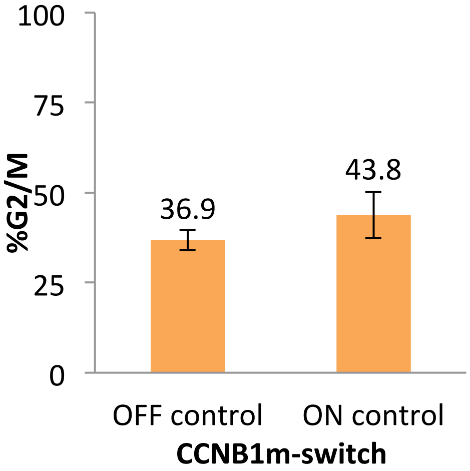

Figure S5: Ribozyme switch controls for arresting cells in G2/M. An OFF control (non-switch, wild-type sTRSV hammerhead ribozyme) and an ON control (non-switch, non-cleaving sTRSV hammerhead ribozyme) were inserted in the 3' UTR of CCNB1m, stably integrated into U2-OS T-Rex Flp-In cells, and tested for their ability to arrest cells in G2/M.

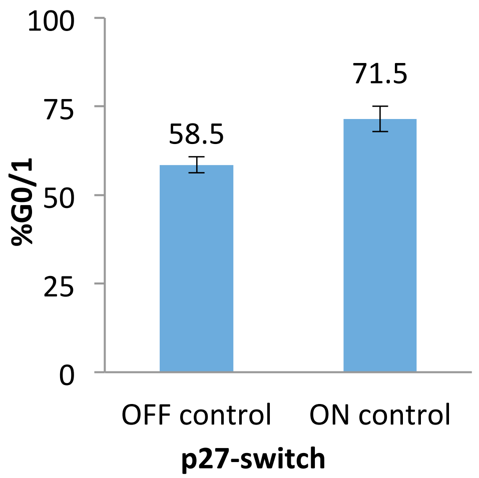

Figure S6: Ribozyme switch controls for arresting cells in G0/1. An OFF control (non-switch, wild-type sTRSV hammerhead ribozyme) and an ON control (non-switch, non-cleaving sTRSV hammerhead ribozyme) were inserted in the 3' UTR of p27, stably integrated into U2-OS T-Rex Flp-In cells, and tested for their ability to arrest cells in G0/1.

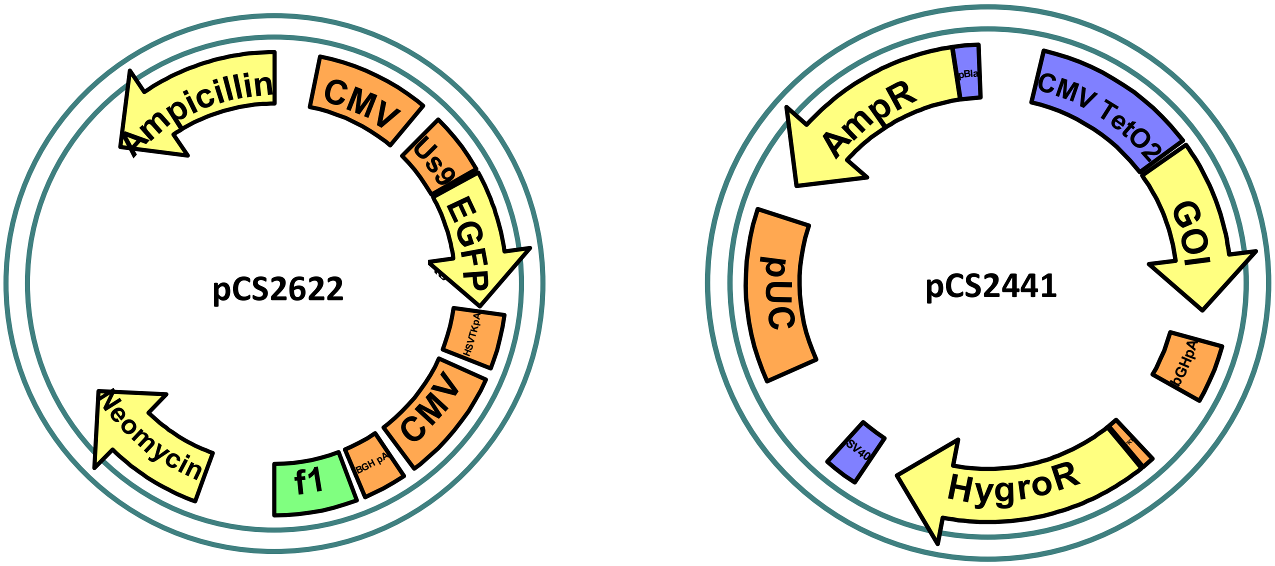

Figure S7: Plasmid maps of the two main plasmid backbones, pCS2622 and pCS2441. For pCS2441, the default gene of interest (GOI), DsRed, is subsequently replaced by sequences encoding regulatory nodes and ribozyme switches (or appropriate control).

Table S1: Regulatory nodes tested in this study. The nodes and a simplified descriptions of their functions in the mammalian cell cycle are listed in alphabetical order. When no reference is provided, see [4] for more information.

Regulatory Node	Function
BubR1	BubR1 is a spindle checkpoint protein involved in the detection of unattached kinetochores. Binding to Cdc20 suppresses anaphase-promoting complex (APC) function and inhibits metaphase-to-anaphase transition.
Cdh1	An activator of the APC such that the active APC-Cdh1 complex recognizes and destroys S and M cyclins and maintain cells in G1. Overexpression shown to arrest mammalian cells in G1[5].
Chk2, Chk1	Chk2,1 receive signaling of DNA damage response kinases ATR and ATM. Chk1 and Chk2 initiate many pathways for DNA repair, cell death or cell cycle arrest. Logically, DNA damage during S or G2 prevents entry into mitosis. Together they phosphorylate and inhibit the activity of the Cdc25 family (A, B, C). Specifically phosphorylate Cdc25C at Ser 216/287, which inhibits Cdc25C phosphatase activity, leading to an increase in inhibitory phosphorylation and therefore deactivation of M-Cdks (Cdk1). Also promotes activity of p53, which increases cell cycle arrest.
Cyclin A	Cyclin A is considered an S-phase cyclin, but has a role in mitosis. Specifically, it is a rate-limiting step for entry into mitosis [6]. Although direct role in arresting G2/M is unclear, because there was potential for inducing arrest in G2/M, cyclin A was tested. Cyclin A1 is expressed in germ cells and early embryo (partner Cdk1). Cyclin A2 (partner Cdk2) has no obvious difference in cell cycle function compared to cyclin A2 and therefore the subtypes are not generally distinguished.
Cyclin B	Cyclin B levels rises at the beginning of mitosis, cyclin B-Cdk activity activates the APC, and is destroyed by APC in late mitosis. Both cyclin B1 and B2 bind Cdk1. Drives centrosome separation, nuclear envelope breakdown and spindle assembly. Cyclin B1 seems to be more important (deletion causes embryonic lethality in mice while deletion of cyclin B2 does not). Complex into the nucleus in late prophase, promotes nuclear envelope breakdown, then redistributes into cytoplasm again after envelope breakdown. Cyclin B1mut (CCNB1(L45A, R42A) is non-degradable [7, 8].
Cyclin D1	Cyclin D-CDk activates E2F-dependent gene expression and progression from G0/1 to S. Here, this serves as a control that should show decreased arrest in G0/1, as supported by the data.
DP-1	Transcription factor that forms a heterodimer with E2F. DP family proteins are one part of a heterodimer with an E2F family protein that forms an E2F complex. These complexes control gene expression of G1/S and S cyclins among other genes.
Emi1	Emi1 inhibits Cdc20. Activated APC(Cdc20) is needed for the meta-phase to anaphase transition. Thus overexpression of Emi1 should result in increased proportion of cells in S and G2/M [5].

Gadd45	GADD45 family of stress response proteins. Is needed in G2/M arrest induced by UV radiation or alkylating agents [9].
hRb	pRB proteins inhibit E2F-dependent gene expression, which activates cyclins that trigger the transition from G1 to S. Therefore overexpression should result in arrest of cells in G0/1. Here, neither overexpression of Rb nor pRBΔCDKHA (not shown) resulted in significant arrest [10]. pRBsm contains 2 nucleotide mutations that results in to Thr > Ala mutations at Thr821 and Thr836 specifically. Phosphorylation at Thr-821 and Thr-826 promotes interaction between the C-terminal domain C and the Pocket domain, and thereby inhibits interactions with heterodimeric E2F/DP transcription factor complexes (genecards.org).
Mad2	Mad2 is a spindle checkpoint component that detects unattached kinetochores. When the checkpoint fails, Mad2 binds Cdc20, inhibiting function
Myc	Myc is a transcription factor that regulates genes involved in metabolism, growth, ribosome synthesis, and differentiation but also division regulatory molecules like cyclin D2 and Cdk4. While most of the gene targets are related to growth rather than directly to division, in certain cell types division is tightly linked to growth.
Myt1	Two inhibitory phosphorylations in Cdk (Thr 14, Tyr 15) and especially the M-Cdks. Wee1 is a kinase that catalyzes the inhibitory phosphorylation at Thr 14 and Myt1 is a kinase that catalyzes the phsophorylation of both Thr 14 and Tyr 15. In theory, if overexpression led to increased activity of either of these enzymes, then M-Cdks would be inhibited and cells would be more often arrested in G2/M.
p16(INK4a)	Inhibitor of Cdk4 and Cdk6. INK4 family only bind Cdk4 and 6, preferring Cdk monomers and increased levels helps disassemble cyclin D-Cdk4,6 complexes. U2-OS, which is deficient in p16 should be especially sensitive to overexpression and this has been shown previously [10]
p21(Cip1/Waf1)	Inhibits cyclin E-Cdk2 and cyclin A-Cdk2,1 (G1/S- and S-Cdks respectively) and activates cyclin D-Cdk4 (G1-Cdks). Cip/Kip family of proteins help assemble the cyclin D-Cdk4,6 complexes, but inhibit the G1/S-Cdks and S-Cdks.
p27(Kip1)	Inhibits cyclin E-Cdk2 and cyclin A-Cdk2,1 (G1/S- and S-Cdks respectively) and activates cyclin D-Cdk4 (G1-Cdks). Cip/Kip family of proteins help assemble the cyclin D-Cdk4,6 complexes, but inhibit the G1/S-Cdks and S-Cdks.
PP2A	PP2A (protein phosphatase type 2A). Enhance inhibitory phosphorylations on M-Cdk by activating Wee1 and inactivating Cdc25 [11].
Wee1	Wee1mut contains S53/123A and E116/117A, meant to reduce de-stabilizing phosphorylations. S53/123 are important for recognition by SKP1/Cul1/F-box protein (SCF) complex with beta-TrCP and phosphorylation leads to degradation [12]. Binding of beta-TrCP was eliminated with E116/117A [12]. These changes should lead to a more stable Wee1 protein, whose overexpression results in increased arrest of cell in G2/M.

Table S2: Ribozyme switch performance in yeast and HEK293. Switch activities are reported as measured by the ratio of GFP/mCherry in a cell population (yeast or HEK293) harboring the indicated switch in the two-color characterization plasmid via flow cytometry.

Switch Name in Legend	Alternative Switch Name	Yeast Basal	Yeast Induced	HEK293 Basal	HEK293 Induced	Source
OFF only	sTRSV	0.051 +/-0.003	0.051 +/-0.003	0.044	0.044	[13]
Switch th-A	theoRs2 OR Theo(A)-AAAAA	0.056 +/-0.002	0.509 +/-0.074			[13]
Switch th-B	theoRs4 OR Theo(A)-CAGAA	0.158 +/-0.006	1.258 +/-0.091			[13]
Switch th-C	L2b9(1x)	0.300	0.720	0.637	0.926	[1]
Switch th-Cx2	L2b9(2x)	n/a	n/a	0.314	0.801	[1]

Table S3: Cell lines created in this study. Cell lines are created by stable integration into U2-OS T-Rex FlpIn lines using the FlpIn system. Blasticidin selection maintains T-Rex integration. Hygromycin selection maintains integrated cassette.

Name in Legend	Plasmid #	Cell Line Description
p27 OFF only	pCS3250	pCS2441-syn21p27-sTRSV
p27 Switch th-A	pCS3252	pCS2441-syn21p27-theoRs2
CCNB1m OFF only	pCS3257	pCS2441-syn21CCNB1mut-sTRSV
CCNB1m Switch th-C	pCS3259	pCS2441-syn21CCNB1mut-L2b9(1x)
CCNB1m Switch th-Cx2	pCS3260	pCS2441-syn21CCNB1mut-L2b9(2x)
CCNB1m Switch th-A	pCS3261	pCS2441-syn21CCNB1mut-theoRs2
CCNB1m Switch th-B	pCS3262	pCS2441-syn21CCNB1mut-theoRs4

Table S4: Primer sequences used in this study.

Name	Sequence
25 ACTB FWD	AGATCAAGATCATTGCTCCTCCT
26 ACTB REV	TCATAGTCCGCCTAGAAGCAT
ApaI_XbaI_XhoI_CMV_rev	AATAGGGCCCCGAGAACTTGTCTAGACGAGAACTTGCTC GAGTGAGCTCTGCTTATATAGACCTCC
ApaI_XhoI_CCNB1_rv	ATATGGGCCCTCTAGCCCGGTCTCGAGTTACACCTTTGC CACAGCCTT
B1m-4_fw	AACAGCTGCTGGGGACATT
B1m-4_rv	ATAGGCTCAGGCGAAAGTTTTT
BamHI_XbaI_CCNB1_fw	ATTAGGATCCTCTAGAAACTTAAAAAAAAAAATCAAAA TGGCGCTCCGAGTCAC
BsrGI_ EcoRI_bGHpA_fwd	AATATGTACAAGTAACGAATTCCTGTGCCTTCTAGTTGC CAG
EGFP_EcoRI_rev	AATAGAATTCTTACTTGTACAGCTCGTCCA

GB_2441_syn21_fw	AGATCGTCGACGGGGTACCGAGCTCGGATCCAACTTAAAAAAAAAAATCAAAATGTCAAACGTGCGAGTGTCTAACG
GB_2441_Wee1mut_fw	GATAGAGATCGTCGACGGGGTACCGAGCTCAACTTAAAAAAAAAAATCAAAATGAGTTTCCTGTCAAGACAGC
GB_2441_Wee1mut_rw	CACAGTCGAGGCTGATCAGCGGGTTTAAACGGGCCCTCTAGCCCGGTCTCGAGCTAGTAGATAGTCAGTGACACGGAC
GB_2622_p27_fw	ATATAAGCAGAGCTCACTCTAGAGCCGCGTAGGGGCGCTTT
GB_2622_p27_rv	GGGTTTAAACGGGCCCTCTAGCCCGGTCTCGAGTTACGTTTGACGTCTTCTGAGGCCAG
GB_2622_RB1_p1_fw	TAAGCAGAGCTCACTCGACTATCGATACCACCATGCCGCCCAAAACCCCC
GB_2622_RB1_p2_rv	AACGGGCCCTACGTCTTGACTCGAGTCATTTCTCTTCCTTGTTTGAGGTATC
GB_Cas9_P2A_fw	CGAAAAAGGCCGGCCAGGCAAAAAAGAAAAAGAAAAAGGCTACTAACTTCAGCCTGCTG
GB_IRES_p27_rv	ACCGAACAAAACAAAGCGCCCCTACGCGGCGATATCGGCCATATTATCATCGTGTTTTTCAAAGGAAAACC
GB_postRZ_rv	TAAGCTTGATCCCTCGATGTTAACTCTAGAGTTTAAACGGGCCCTCTAGACTCGA
GB_RB1_p2p1_fw	AGGTCTGCCAGCACCAACAAAAATGGCTCCAAGATCAAGAATCTTAGTATCAATTGGTG
GB_RB1_p2p1_rv	TTTGTTGGTGCTGGCAGACCTTCTGAAATTTTATATGGACTC
HSVTK_EcoRI_fwd	AATAGAATTCATGAGACGATCTCATGCTGGA
HSVTK_EcoRV_rev	AATAGATATCTACTGAGAGTGCACCATAGGG
MCS_fwd	phosph–AATTGTCGCGAGCTAGCCTTAAGAAGCTTCCTAGGC
MCS_rev	phosph–CCGGGCCTAGGAAGCTTCTTAAGGCTAGCTCGCGAC
p16_ ApaI_rv	ATAAGGGCCCTACGTCTTGACTCGAGTCAATCGGGGATATCT
p16_SalI_XbaI_fw	AATAGTCGACTCTAGAACCACCATGGAGCCTTC
RzApaIPacI_rv	AGAAGGGCCCAAGATTAATTAAAAAAAAAATTTTTATTTTTCTTTTTGCTGTT
RzApaIPacI_rv	AGAAGGGCCCAAGATTAATTAAAAAAAAAATTTTTATTTTTCTTTTTGCTGTT
RzXhoIAsiSI_fw	AATACTCGAGGCGATCGCAAACAAACAAAGCTGTCACCG
RzXhoIAsiSI_fw	AATACTCGAGGCGATCGCAAACAAACAAAGCTGTCACCG
Us9_KpnI_fwd	AATAGGTACCATGCCCGCCGC

Table S5: List of the plasmids used in this study. Plasmids obtained from Addgene are indicated by the Addgene plasmid number.

Plasmid #	Addgene #	Description	Reference
pCS2052	18657	pBB14	[2]
pCS2622		CMV-Us9-EGFP	this work
pCS2441		CMVTetO2-DsRed-bGHpA (FlpIn)	this work
pCS2587		CMV-mCherry	gift from Melina Mathur
pCS2623	10720	pSG5L HA RB	[14]
pCS2624	10916	pCMV p16 INK4A	[15]
pCS2625	11596	HA-cdh1	[16]
pCS2626	37968	pCMV-Neo-Bam DP1	[17]
pCS2770		ECE-pRBΔCDK-HA	[10]
pCS2772	11182	pCDNA-cyclinD1HAT286A	[18]
pCS2773	16240	Flag p21 WT	[19]
pCS2774	14049	pCMV5 human p27	[20]
pCS3245		pCS2622-pRBsm	this work
pCS3341		pCS2622-p16	this work
pCS3342		pCS2622-p27	this work
	47330	BubR1	[21]
	16047	Mad2	[22]
	41901	Chk2	[23]
	39854	Emi1	[24]
	22894	Chk1	[25]
	24929	GADD45	[26]
	22695	pMyt1a	[27]
	10911	CyclinB2	[28]
	26060	CyclinB1	[29]
	8959	CyclinA	[28]
pCS3246		pCS2441-Wee1mut	this work
	39856	Myc	[30]
	15248	PP2A	[31]
	39852	CyclinB1mut	[7]
pCS3249		pCS2441-syn21p27	this work
pCS3247		pCS2786-Cas9-mCherry-theoRs2	this work
pCS3250		pCS2441-syn21p27-sTRSV	this work
pCS3252		pCS2441-syn21p27-theoRs2	this work
pCS3256		pCS2441-syn21CCNB1mut	this work
pCS3257		pCS2441-syn21CCNB1mut-sTRSV	this work
pCS3259		pCS2441-syn21CCNB1mut-L2b9(1x)	this work
pCS3260		pCS2441-syn21CCNB1mut-L2b9(2x)	this work

pCS3261		pCS2441-syn21CCNB1mut-theoRs2	this work
pCS3262		pCS2441-syn21CCNB1mut-theoRs4	this work

Table S6: Sequences of ribozyme controls and switches used in this study. Sequences listed below include the restriction sites for XhoI (ctcgag) and AsiSI (gcgatcgc) 5' of the switch sequence and PacI (ttaattaa) and ApaI (gggcccc) 3' of the sequence. Main switch sequence is underlined and flanked by short spacer sequences which are italicized.

Switch Name in Legend	Alternative Switch Name	Sequence	Source
OFF only	sTRSV	ctcgaggcgatcgc*AAACAAACAAA*GCTGTCACCGGATGTGCTTTCCGGTCTGATGAGTCCGTGAGGACGAAACAGC*AAAAAGAAAAATAAAAATTTTTT*GGAAgggcccc	[13]
Switch th-A	theoRs2 OR Theo(A)-AAAAA	ctcgaggcgatcgc*AAACAAACAAA*GCTGTCACCGGAATACCAGCATCGTCTTGATGCCCTTGGAAGTCCGGTCTGATGAGTCCAAAAAGGACGAAACAGC*AAAAAGAAAAATAAAAATTTTTTTT*ttaattaaTCTTgggcccc	[13]
Switch th-B	theoRs4 OR Theo(A)-CAGAA	ctcgaggcgatcgc*AAACAAACAAA*GCTGTCACCGGAATACCAGCATCGTCTTGATGCCCTTGGAAGTCCGGTCTGATGAGTCCCAGAAGGACGAAACAGC*AAAAAGAAAAATAAAAATTTTTTTT*ttaattaaTCTTgggcccc	[13]
Switch th-C	L2b9(1x)	ctcgaggcgatcgc*AAACAAACAAA*GCTGTCACCGGATGTGCTTTCCGGTCTGATGAGTCCGTTGTCCAATACCAGCATCGTCTTGATGCCCTTGGCAGTGGATGGGGACGGAGGACGAAACAGC*AAAAAGAAAAATAAAAATTTTTTTT*ttaattaaTCTTgggcccc	[1]
Switch th-Cx2	L2b9(2x)	ctcgaggcgatcgc*AAACAAACAAA*GCTGTCACCGGATGTGCTTTCCGGTCTGATGAGTCCGTTGTCCAATACCAGCATCGTCTTGATGCCCTTGGCAGTGGATGGGGACGGAGGACGAAACAGC*AAAAAGAAAAATAAAAATTTTTTTT*TTAATCGC*AAACAAACAAA*GCTGTCACCGGATGTGCTTTCCGGTCTGATGAGTCCGTTGTCCAATACCAGCATCGTCTTGATGCCCTTGGCAGTGGATGGGGACGGAGGACGAAACAGC*AAAAAGAAAAATAAAAATTTTTTTT*ttaattaaTCTTgggcccc	[1]

REFERENCES

1. Wei KY, Chen YY, Smolke CD. A yeast-based rapid prototype platform for gene control elements in mammalian cells. Biotechnology and bioengineering. 2013;110(4):1201-10. doi:10.1002/bit.24792.

2. Brideau AD, Banfield BW, Enquist LW. The Us9 gene product of pseudorabies virus, an alphaherpesvirus, is a phosphorylated, tail-anchored type II membrane protein. Journal of virology. 1998;72(6):4560-70.

3. Bloom RJ, Winkler SM, Smolke CD. A quantitative framework for the forward design of synthetic miRNA circuits. Nature methods. 2014;11(11):1147-53. doi:10.1038/nmeth.3100.

4. Morgan DO. The cell cycle : principles of control. Primers in biology. London
Sunderland, MA: Published by New Science Press in association with Oxford University Press ; Distributed inside North America by Sinauer Associates, Publishers; 2007.

5. Hsu JY, Reimann JD, Sorensen CS, Lukas J, Jackson PK. E2F-dependent accumulation of hEmi1 regulates S phase entry by inhibiting APC(Cdh1). Nat Cell Biol. 2002;4(5):358-66. doi:10.1038/ncb785
ncb785 [pii].

6. Pagano M, Pepperkok R, Verde F, Ansorge W, Draetta G. Cyclin A is required at two points in the human cell cycle. EMBO J. 1992;11(3):961-71.

7. Gavet O, Pines J. Activation of cyclin B1-Cdk1 synchronizes events in the nucleus and the cytoplasm at mitosis. The Journal of cell biology. 2010;189(2):247-59. doi:10.1083/jcb.200909144.

8. Gavet O, Pines J. Progressive activation of CyclinB1-Cdk1 coordinates entry to mitosis. Dev Cell. 2010;18(4):533-43. doi:S1534-5807(10)00111-5 [pii]

10.1016/j.devcel.2010.02.013.

9. Wang XW, Zhan Q, Coursen JD, Khan MA, Kontny HU, Yu L et al. GADD45 induction of a G2/M cell cycle checkpoint. Proc Natl Acad Sci U S A. 1999;96(7):3706-11.

10. Lukas J, Herzinger T, Hansen K, Moroni MC, Resnitzky D, Helin K et al. Cyclin E-induced S phase without activation of the pRb/E2F pathway. Genes Dev. 1997;11(11):1479-92.

11. Janssens V, Goris J. Protein phosphatase 2A: a highly regulated family of serine/threonine phosphatases implicated in cell growth and signalling. Biochem J. 2001;353(Pt 3):417-39.

12. Watanabe N, Arai H, Nishihara Y, Taniguchi M, Hunter T, Osada H. M-phase kinases induce phospho-dependent ubiquitination of somatic Wee1 by SCFbeta-TrCP. Proc Natl Acad Sci U S A. 2004;101(13):4419-24. doi:10.1073/pnas.0307700101
0307700101 [pii].

13. Townshend B, Kennedy AB, Xiang JS, Smolke CD. High-Throughput, Data-Rich Cellular RNA Device Engineering. Nature methods. 2015.

14. Sellers WR, Novitch BG, Miyake S, Heith A, Otterson GA, Kaye FJ et al. Stable binding to E2F is not required for the retinoblastoma protein to activate transcription, promote differentiation, and suppress tumor cell growth. Genes Dev. 1998;12(1):95-106.

15. Medema RH, Herrera RE, Lam F, Weinberg RA. Growth suppression by p16ink4 requires functional retinoblastoma protein. Proc Natl Acad Sci U S A. 1995;92(14):6289-93.

16. Pfleger CM, Lee E, Kirschner MW. Substrate recognition by the Cdc20 and Cdh1 components of the anaphase-promoting complex. Genes Dev. 2001;15(18):2396-407. doi:10.1101/gad.918201.

17. Moberg K, Starz MA, Lees JA. E2F-4 switches from p130 to p107 and pRB in response to cell cycle reentry. Molecular and cellular biology. 1996;16(4):1436-49.

18. Newman RM, Mobascher A, Mangold U, Koike C, Diah S, Schmidt M et al. Antizyme targets cyclin D1 for degradation. A novel mechanism for cell growth repression. The Journal of biological chemistry. 2004;279(40):41504-11. doi:10.1074/jbc.M407349200.

19. Zhou BP, Liao Y, Xia W, Spohn B, Lee MH, Hung MC. Cytoplasmic localization of p21Cip1/WAF1 by Akt-induced phosphorylation in HER-2/neu-overexpressing cells. Nat Cell Biol. 2001;3(3):245-52. doi:10.1038/35060032.

20. Luo Y, Hurwitz J, Massague J. Cell-cycle inhibition by independent CDK and PCNA binding domains in p21Cip1. Nature. 1995;375(6527):159-61. doi:10.1038/375159a0.

21. Holland AJ, Fachinetti D, Han JS, Cleveland DW. Inducible, reversible system for the rapid and complete degradation of proteins in mammalian cells. Proc Natl Acad Sci U S A. 2012;109(49):E3350-7. doi:10.1073/pnas.1216880109.

22. Wassmann K, Liberal V, Benezra R. Mad2 phosphorylation regulates its association with Mad1 and the APC/C. EMBO J. 2003;22(4):797-806. doi:10.1093/emboj/cdg071.

23. Matsuoka S, Ballif BA, Smogorzewska A, McDonald ER, 3rd, Hurov KE, Luo J et al. ATM and ATR substrate analysis reveals extensive protein networks responsive to DNA damage. Science. 2007;316(5828):1160-6. doi:10.1126/science.1140321.

24. Di Fiore B, Pines J. Emi1 is needed to couple DNA replication with mitosis but does not regulate activation of the mitotic APC/C. The Journal of cell biology. 2007;177(3):425-37. doi:10.1083/jcb.200611166.

25. Unsal-Kacmaz K, Chastain PD, Qu PP, Minoo P, Cordeiro-Stone M, Sancar A et al. The human Tim/Tipin complex coordinates an Intra-S checkpoint response to UV that slows replication fork displacement. Molecular and cellular biology. 2007;27(8):3131-42. doi:10.1128/MCB.02190-06.

26. Yang Q, Manicone A, Coursen JD, Linke SP, Nagashima M, Forgues M et al. Identification of a functional domain in a GADD45-mediated G2/M checkpoint. The Journal of biological chemistry. 2000;275(47):36892-8. doi:10.1074/jbc.M005319200.

27. Kim JG, Hudson LD. Novel member of the zinc finger superfamily: A C2-HC finger that recognizes a glia-specific gene. Molecular and cellular biology. 1992;12(12):5632-9.

28. Hinds PW, Mittnacht S, Dulic V, Arnold A, Reed SI, Weinberg RA. Regulation of retinoblastoma protein functions by ectopic expression of human cyclins. Cell. 1992;70(6):993-1006.

29. Hagting A, Jackman M, Simpson K, Pines J. Translocation of cyclin B1 to the nucleus at prophase requires a phosphorylation-dependent nuclear import signal. Current biology : CB. 1999;9(13):680-9.

30. den Elzen N, Pines J. Cyclin A is destroyed in prometaphase and can delay chromosome alignment and anaphase. The Journal of cell biology. 2001;153(1):121-36.

31. Sablina AA, Chen W, Arroyo JD, Corral L, Hector M, Bulmer SE et al. The tumor suppressor PP2A Abeta regulates the RalA GTPase. Cell. 2007;129(5):969-82. doi:10.1016/j.cell.2007.03.047.
